# Treatment of Pyorrhea Alveolaris

**Published:** 1884-06

**Authors:** A. W. Harlan


ARTICLE IV.

### TREATMENT OF PYORRHEA ALVEOLARIS.

PAPER BY A W. HARLAN, CHICAGO.

On account of the uncertainty of the mind of the dental specialist with reference to the treatment of that variously named and idly defined disease, known as pyorrhea alveolaris, Riggs' disease, alveolitis, phagedena pericementii, etc., the writer desires to offer a few suggestions relating thereto, which may tend to give more confidence to those who have had but little success in their efforts to restore to health the tissues which are so often ravaged beyond repair. It will not be necessary in this report to say anything of the etiology of the disease under discussion, beyond the expression of the belief that it is infectious, and that micro organisms are at least partially responsible for the disastrous results which are sure to follow if it is not checked before the complete destruction of the alveoli.

It is now nearly two years since I became convinced that the methods of treatment advocated by various writers, including that of Dr. Riggs himself, were not the most beneficial to the majority of cases presenting themselves. At that time I was engaged in making some experiments with the essential oils and eucalyptus globulus, to determine their value as antiseptics. During these investigations it occurred to me that iodide of zinc, which then, as it does now, occupied only a few lines in the "Dispensatory" and works on medical chemistry and therapeutics, might be the remedy which would rob the hydra-headed monster of its terrors for the dental surgeon. I procured a sample and at once made solutions of xii, and xxiv, grs, to the ounce of water, and waited for the first case. It soon came. My first experience with it was in the fall of 1881, and the first case was that of a lower central incisor; the gum was loose two-thirds of the length of the root on the labial surface, pus exuding on pressure, and the alveolar process wasted half the length of the root; there was little, if any, deposit on the lacial surface of the root, and no salivary calculus on any of the teeth, except the buccal surfaces of the superior third molars. I carefully cleansed the root and excised the edge of the bone, then dried the pocket, after washing it out with water, and injected into it three drops of the first-named solution. The patient was directed not to squeeze the gum, but to return in four days, and let me see him. When he returned, I dried the gums and applied pressure, and found scarcely any discharge. I repeated the former treatment, omitting the scraping and probing, and when he returned the second time there was no discharge whatever. The pocket had commenced to fill up, and the gum was beginning to re-attach itself to the tooth. The patient returned again after eight days, and there remained hardly a trace of the disease. To all intents and purposes it was cured. After the lapse of more than twenty months the gum remains firm, and is of normal color, without any external evidence of having been diseased at all. The remarkable manner in which this remedy exerted its beneficial influence in this case caused me to practically discard all other therapeutical agents in the treatment of pyorrhea, except as shall be hereinafter mentioned. I found from a study of cases when a patient was suffering from an acute attack of pyorrhea the pockets should first be packed with iodoform and eucalyptus, iodoform and oil of cinnamon, or be thoroughly syringed with a one to three-grain solution to the ounce of water, of chloride of alumina, which is a good disinfectant and astringent, and also an excellent bleacher for discolored, pulpless teeth; but the method of using it in such cases will be dwelt upon in the discussion on operative dentistry. This method of treating the pockets in acute cases relieves the patient of present suffering, and also reduces the swollen gums to their normal size. In three or four days the sanguinary deposits may be removed and the edges of the alveoli scraped or burred off. The pockets should then be syringed with peroxide of hydrogen, which will cleanse them perfectly, and at the same time bring into direct contact with the diseased pocket a germ-destroyer and antiseptic not less potent than any which can be named. After drying the gums, the pockets should not be injected with the previously named solution of iodide of zinc, one, two, or three drops, or more, in each pocket. When the patient returns on the fourth day the gums must be dried carefully, and a fine cone of cotton or bibulous paper be moistened with peroxide of hydrogen and gently pressed into each pocket. If there should be any effervescence, it demonstrates the presence of pus, and each pocket must be again injected with the iodide of zinc solution. Peroxide of hydrogen, for all clinical purposes, is a perfect detector of pus, even better than the microscope, because those not familiar with it can see with the naked eye the bubbling produced by $H_2O_2$, when brought into contact with pus.

In chronic cases, after the removal of the diseased bone surgically and the cleansing of the roots are effected, the pockets should be syringed with $H_2 O_2$, to be followed by the injection of the xxiv., gr. solution of iodide of zinc in precisely the same manner as has been heretofore described. In very bad or almost hopeless cases, I use even a stronger solution, grs. xlviii, to the ounce; and when the gingival margins present a ragged border or cone-shaped slit, I apply the pure granular iodide to the edges of the slit once in three days, and soon find a perfect restoration of the normal festooning of the gingeval margin. The injection into the pocket is to be repeated every fourth day. The length of time required for the cure of cases where two to four or five teeth are involved, varies from twelve days to four or five weeks, supposing the patient to be in ordinary good health. In those cases where constitutional treatment is required each must be met according to the indications; and the intelligent practitioner will decide for himself just what is needed during the period of local medication. The worst case that I have had during the past two years was where sixteen teeth were diseased, and it required constant care from March 26th, to June 12th, 1883. It was a case of three years' standing, and had been under the care of two gentlemen before it came to me, and the patient thought her gums were worse after leaving them than before treatment was commenced.

Iodide of zinc has long been used by medical practitioners, in the form of a syrup for internal administration, and as an ointment externally for strumous inflammation and enlargements, chorea, etc. It is made by digesting four parts of iodine with a little more than one part of zinc and twenty parts of water, until the liquid has become colorless. On evaporation it crystalizes and is ready for use. It is freely soluble in water. I present herewith a comparative table of the relative value of antiseptics and germ-destroyers, which as all may see places iodine almost at the head of the list.

This table is the result of a large number of experiments by M. Miguel, of the Observatoire Montsouris, and shows the minimum quantity of the several antiseptics capable of preventing the development of germs and of adult bacteria in a liter of bouillon :

| | Gms. | | Gms |
|---|---|---|---|
| Peroxide of hydrogen | 0.05 | Arsenious acid | 6.00 |
| Bichloride of mercury | 0.07 | Sulphate of strychnine | 7.00 |
| Iodine | 0.25 | Boracic acid | 7.50 |
| Chloride of gold | 0.25 | Arsenite of soda | 9.00 |
| Bichloride of platinum | 0.30 | Hydrate of chloral | 9.30 |
| Cyanhydric acid | 0.40 | Salicylate of soda | 10.30 |
| Bromide | 0.60 | Caustic soda | 18.00 |
| Chloroform | 1.00 | Borate of soda | 70.00 |
| Bichromate of potassa | 1.20 | Chlorhydrate of morphine | 75.00 |
| Ammoniacal gas | 1.40 | Alcohol | 75.00 |
| Thymic acid | 2.00 | Iodide of potassium | 150.00 |
| Phenic [carbolic] acid | 3 20 | Marine salt | 165.00 |
| Permanganate of potassa | 3.50 | Glycerine | 225.00 |
| Acetate of lead | 3.60 | Sulphate of ammonia | 250 00 |
| Alum | 4.50 | Hyposulphite of soda | 275.00 |
| Bromo-hydrate of of quinine | 5.50 | | |

This system of treatment, based on the primary injection of $H_2O_2$ into the pockets as a ready method of cleansing them, and at the same time furnishing nascent oxygen to destroy the unclassified micro-organisms there present, and the subsequent injection of the zinc iodide, for it is well known stimulating and protective effects on the reparative material exuded, which is always ready for organization after being thus carefully protected, renders it almost certain that if careless probing or syringing of the pockets is prohibited, and the mouth is well cleansed with an antiseptic wash, a reproduction of lost tissues may be confidently anticipated.

In conclusion, permit me to say that this remedy, when used faithfully in conjunction with peroxide of hydrogen, the most valuable germ destroyer known up to the present time, arms us so well that I believe with expert handling of the probe to discover the concealed pocket, skillful removal of all deposits and edges of diseased bone, conjoined to an exact knowledge of constitutional needs, that we no longer need feel dismayed when brought face to face with a genuine case of pyorrhea alveolaris.—*Transactions American Dental Association.*

